# The Childbirth Experience of Pregnant Women Living with HIV Virus

**DOI:** 10.3390/jcm14061975

**Published:** 2025-03-14

**Authors:** Andréa Paula de Azevedo, Luisa Castro, Cristina Barroso Hofer, Francisca Rego

**Affiliations:** 1 Faculty of Medicine, University of Porto, 4200-319 Porto, Portugal; luisacastro@med.up.pt (L.C.); mfrego@med.up.pt (F.R.); 2 Martagão Gesteira Childhood and Pediatric Institute, Federal University of Rio de Janeiro, Rio de Janeiro 21941-612, Brazil; 3 Center for Health Technology and Services Research (CINTESIS@ RISE), Faculty of Medicine, University of Porto, 4200-319 Porto, Portugal; 4 Department of Infectious Diseases, Federal University of Rio de Janeiro (UFRJ), Rio de Janeiro 21941-630, Brazil

**Keywords:** childbirth, HIV, obstetrics, patient satisfaction, questionnaires

## Abstract

**Objective:** The aim of this study was to examine the childbirth satisfaction of pregnant women living with HIV virus (PWLWHIV) and its association with selected variables in order to improve it. **Methods:** A total of 82 PWLWHIV were interviewed at an Institute in Rio de Janeiro. Maternal satisfaction was measured using the Brazilian version of the Mackey Childbirth Satisfaction Rating Scale, which was translated to Brazilian Portuguese by Lopes, who validated the translated version. The Mackey scale is divided into six subscales: self-evaluation, partner, baby, midwives, doctors, and overall satisfaction. **Results:** The PWLWHIV experienced a good total childbirth satisfaction (score 133 out of 165) and good overall childbirth satisfaction (score 12 out of 15). The PWLWHIV also experienced a good self-satisfaction (score 37.3 out of 45), good satisfaction with partner (score 8.96 out of 10), good satisfaction with midwives (score 35.1 out of 45), and good satisfaction with doctors (score 31.2 out of 45). The PWLWHIV also experienced satisfaction with their babies (score 8.48 out of 10). Significant associations were found through univariable and multiple regression analysis, regarding complications with baby at birth (*p* < 0.001) on the total, overall, self, partner, midwife, and doctor subscales. **Conclusions:** PWLWHIV had a positive experience at childbirth in the public maternities units of Rio de Janeiro. Our findings indicate that complications regarding baby health at birth were the main factor associated with a negative experience.

## 1. Objective

The aim of this study was to examine the childbirth satisfaction of pregnant women living with HIV (PWLWHIV) and its association with selected variables in order to improve it.

## 2. Introduction

Maternal HIV (human immunodeficiency virus) infection is an important global health concern with many repercussions on perinatal outcomes [1]. Maternal HIV infection increases the risk of preterm birth, low birth weight, and small for gestational age newborns [1]. Additionally, maternal HIV infection has been linked to higher rates of preeclampsia, particularly in women receiving highly active antiretroviral therapy [2]. The prevalence of pregnant women living with HIV virus (PWLWHIV) varies globally and regionally. Globally, HIV prevalence among pregnant women is estimated at 2.9% (95% CI, 2.4–3.4%) [3]. In Brazil, the HIV prevalence among pregnant women is generally less than 1% [4]. A national hospital-based study conducted between 2011 and 2012 found a prevalence of 0.4% [4]. Another study conducted in 2011 in public health centers in the central-west region of Brazil reported a prevalence of 1.59 cases per 1000 women [5]. These data indicate that, although the HIV prevalence among pregnant women in Brazil is relatively low, there are still significant regional variations according to PWLWHIV access to health services (between 0.2% to 1.59%) [5]. In sub-Saharan Africa, a systematic review and meta-analysis estimated the average HIV incidence rate among pregnant and breast-feeding women to be 3.6 per 100 person-years [6]. Another meta-analysis reported a pooled HIV incidence showing a substantial risk of HIV acquisition during pregnancy, particularly in high-prevalence settings [6,7].

Childbirth is a deeply significant and unique event for pregnant women living with HIV (PWLWHIV), marking an important milestone in their personal journey. As such, evaluating women’s satisfaction with childbirth has become increasingly relevant to governments and healthcare providers, as it serves as a vital indicator of the quality of care received by the mother [8]. The experience of childbirth is crucial not only for the mother’s well-being but also for fostering a strong bond between mother and baby [9,10]. A positive childbirth experience can strengthen this relationship, while traumatic experiences during labor and delivery can have lasting effects. For example, traumatic childbirth experiences may contribute to postpartum depression and post-traumatic stress disorder (PTSD) symptoms, which in turn can negatively impact mother–child interactions and bonding [11,12].

Given the potential emotional and psychological effects of childbirth, particularly for PWLWHIV, it is important for healthcare systems to provide not only physical care but also emotional and psychological support. This can help mitigate negative outcomes and promote healthier mother–baby relationships, ultimately improving both maternal and infant health [13,14].

A positive childbirth experience leaves an inheritance of all the good sensations and long-term memories of the pregnant woman, including emotions, a sense of well-executed work, competence, confidence and decision-making power [15]. The satisfaction of pregnant women with childbirth is influenced by various factors such as the actual outcomes of their experiences, their pre-existing expectations, and the discrepancies between what they anticipated and what they actually experienced [16]. For pregnant women living with HIV (PWLWHIV), additional factors come into play that affect their childbirth satisfaction. Key factors include the quality of care provided during childbirth, the level of support from healthcare professionals, management of HIV-related concerns, and the degree of autonomy that PWLWHIV experience during labor and delivery. The pregnant woman’s perception of safety is identified as one of the key factors influencing childbirth satisfaction [17].

The satisfaction of PWLWHIV at childbirth is influenced by several factors, including the quality of care received at childbirth, the level of support from healthcare professionals, the management of HIV-related concerns, and the autonomy of PWLWHIV [18,19,20]. A significant challenge for PWLWHIV is the HIV-related stigma, which can manifest as shame and discrimination. This stigma negatively impacts their childbirth experience by reducing their willingness to disclose their HIV status, decreasing adherence to medical care, and limiting their use of skilled childbirth services. To improve both maternal and neonatal health outcomes, it is vital to address these issues by implementing stigma reduction strategies and providing supportive, non-discriminatory care.

HIV-related stigma, including shame and discrimination, negatively impacts the childbirth experience of PWLWHIV by reducing disclosure, adherence to care, and utilization of skilled childbirth services. Addressing these issues through stigma reduction interventions and supportive care is crucial for improving maternal and neonatal health outcomes [21,22,23].

The evaluation of women’s satisfaction with childbirth is crucial for enhancing obstetric care and ensuring a positive experience for both mothers and newborns [17]. While patient satisfaction is inherently challenging to measure, assessing women’s experiences of childbirth is essential to gauge the quality of care provided [16]. This process requires reliable and valid tools to accurately measure satisfaction [12,24]. The instruments available for measuring satisfaction are highly varied and multidimensional, making it essential that these tools be tested and validated to ensure their reliability and relevance [25]. Reliability refers to a tool’s ability to produce consistent results over time, while validity ensures that the instrument accurately measures what it is intended to—in this case, women’s satisfaction with childbirth—rather than any related but unintended construct. Despite the availability of various tools for assessing satisfaction, there is considerable variability in their content and quality. Therefore, it is vital to evaluate and standardize these instruments to ensure that they are both reliable and valid, ultimately improving the quality of maternal care [25,26]. The tools used to measure women’s satisfaction with childbirth are diverse and multidimensional, reflecting the complex nature of the childbirth experience. These instruments vary widely in their content, their focus, and the dimensions they measure, which makes it crucial for them to be tested and validated consistently.

By thoroughly assessing and validating these instruments, healthcare providers can use them more effectively to gather accurate feedback from women, leading to improvements in the quality of maternal care and a better understanding of the factors that contribute to satisfaction during childbirth [16,27].

The Mackey Childbirth Satisfaction Rating Scale [28]. is one of the most commonly used instruments for assessing women’s satisfaction with childbirth, and it has been utilized in various countries, including the USA, the United Kingdom, the Netherlands, and Belgium [26,29,30,31].

Researchers have consistently tested the internal consistency of the scale using Cronbach’s alpha, a measure of reliability. However, only three adaptations of the scale—two in Spain [32,33] and one in Iran [34]—have involved in-depth explorations of the factor structure and validation of the psychometric properties of their subscales. This thorough evaluation enhances the tool’s usability and interpretability.

Overall, the Mackey Childbirth Satisfaction Rating Scale demonstrates good validity and reliability for measuring satisfaction in women who have given birth to healthy newborns through vaginal delivery following a full-term pregnancy. Despite its effectiveness, the scale’s main limitation is its length, with over 30 items, which can potentially lead to participant fatigue and reduce ease of use [35].

As the MCSRS had already been translated and validated into Brazilian Portuguese [36,37], we used it for evaluating the childbirth satisfaction of PWLWHIV.

## 3. Material and Methods

This was a prospective observational study conducted in a public prenatal care center of a public Childhood and Pediatrics Institute of Rio de Janeiro which cares for PWL-WHIV. At this institution, childbirth care is provided by doctors who are specialists in obstetrics and gynecology, infectiologists, and nurses. The PWLWHIV participated in the study twice: firstly, before they gave birth, when the sociodemographic questionnaire was applied, and secondly, after they gave birth when the birth and Mackey questionnaire (MCSRS) adapted to Brazil was applied. All the questionnaires was performed by the researcher, and they were filled out in a calm setting without interruptions. The researcher remained available to answer any questions and to clarify any specific aspects of the questionnaire items. The doctors or midwives who provided the assistance were not informed of the study to avoid any bias.

Lopes and Nomura [36,37], who adapted the MCSRS for Brazil, authorized its use in this study.

The inclusion criteria were PWLWHIV 18 years old or older with gestational ages between 20 and 36 weeks, without anomalies.

The exclusion criteria were abortion, preterm birth, fetal death, and PWLWHIV who were not capable of understanding informed consent or the questionnaires. No PWL-WHIV had been excluded in this study by those criteria.

### 3.1. Sample Size

Considering a total population of 776 pregnant women living with HIV at Rio de Janeiro based on the prevalence of PWLWHIV at Rio de Janeiro annually (N), we defined a margin of error of 4 points (resulting in a confidence interval with an amplitude of 8 points), a confidence level of 95%, and a variance of 369 [36,37]. With these parameters, the calculation indicated a minimum sample of 80 participants. Therefore, we set a minimum sample size of 80 pregnant women for the study. For this study, all PWLWHIV that met the inclusion criteria were included until the minimum size was achieved, from June 2023 until December 2023.

### 3.2. Instruments

Sociodemographic data were collected through direct interviews. These included variables such as age, parity, how they self-identified ethnically, whether the woman was living with her partner, educational level, religious belief, habits, and any substance use issues, whether there was a companion at birth, and whether they received prenatal care.

The birth questionnaire was performed through a face-to-face interview. It encompassed the subjective level of pain, the weight and length of the baby, any complications in delivery, and the presence of a partner.

The Mackey Childbirth Satisfaction Rating Scale (MCSRS) is a 34-item scale measuring childbirth satisfaction. It contains five subscales representing the behaviors of the major participants in the event (self, nine items (Q3–Q11); partner, two items (Q12 and Q13); baby, three items (Q14–Q16); nurse, nine items (Q17, Q19, Q21, Q23, Q25, Q27, Q29, Q31, and Q33); physician, eight items (Q18, Q20, Q22, Q24, Q26, Q28, Q30, and Q32)); and one subscale for global overall labor and delivery evaluation (three items (Q1, Q2, and Q34)) [26]. In conformity with the 5-point Likert scale, each item had five choices for responses: (a) very dissatisfied; (b) dissatisfied; (c) neither satisfied nor dissatisfied; (d) satisfied; and (e) very satisfied. The final score is simply the sum of the respondent’s scores (0–165). The scores represent a woman’s perception of the importance of the major participants (self, partner, baby, nurses, and doctors) involved in the process and of her childbirth experience overall. The overall score is the sum of the individual scores. High overall scores indicate total or near-total satisfaction with the childbirth experience. Face and construct validity and reliability were established [8,26,35,37].

The total MCSRS score refers to the sum of all the subscales: self, partner, baby, nurses, doctors, and overall labor.

The Brazilian version of the MCSRS contains six subscales, five of which are the same as those of the Mackey questionnaire, and four further questions asking the pregnant women for their opinions about their childbirth. The MCSRS tool was translated to Brazilian Portuguese and culturally adapted to Brazil, and the resulting version was validated [8,35,37]. The psychometric properties of the Brazilian version of the MCSRS yielded two dimensions related to professional care giving and family [35]. For the factors regarding professional care—satisfaction with nurses, doctors, and the baby, and the overall experience—the Cronbach’s alpha and McDonald’s omega were both 0.96. For the factors concerning the family—self-satisfaction and satisfaction with one’s partner—the alpha and omega were 0.89 and 0.92, respectively. The reliability coefficient omega for the overall reliability of the questionnaire was 0.97. The Brazilian version by Lopes [35] demonstrated good reliability and is thus a potential instrument for promoting improvements in childbirth care in the country [35]. In Spain, the Cronbach’s alpha coefficient was 0.94 for the total scale, ranging from 0.72 to 0.96 for the subscales [33]. In Iran, the Cronbach’s alpha for the total scale was 0.78. It ranged between 0.70 and 0.86 for five subscales and was 0.31 for the “baby” subscale [34]. In our study, the Cronbach’s alphas were 0.990 and 0.977 for the professional care factor and the family factor, respectively.

### 3.3. Data Analysis

The variables were characterized as follows: maternal age (years); parity (primiparous or multiparous); ethnicity (white, mixed race, or black); marital status (single, married, divorced, or consensual union); educational level (no or incomplete high school education, completed high school education, or college education); the use of illicit drugs; smoking; alcohol consumption; occupational status (unemployed or employed); monthly income; planned pregnancy; desired pregnancy; painful delivery; type of delivery (vaginal or cesarean); the newborn’s sex (male or female); first baby; gestational age at birth (weeks); birthweight (grams); 1 min Apgar score (0 = score > 7; 1 = score ≤ 7); length at birth (cm); and complications during birth.

The distributions of the continuous variables were graphically studied, and descriptive statistics for the quantitative variables are presented as means and standard deviations (SDs) for normally distributed quantitative variables and medians and interquartile intervals [IQIs] are presented otherwise. Categorical variables are described as absolute (n) and relative (%) frequencies. Non-normally distributed quantitative variables were compared using the Mann–Whitney U test.

A multiple regression analysis was conducted on the patient sample to identify which variables were associated with the following outcomes: MCSRS Overall Global Satisfaction; MCSRS Self; MCSRS Partner; MCSRS Baby; MCSRS Midwives; MCSRS Doctors; MCSRS Total Childbirth Satisfaction. Initially, all the relevant variables were tested using simple linear regression models for each outcome. Next, variables that were significant at the *p* < 0.2 level in the simple models were included in the initial multiple models of each outcome. The final model of each outcome was obtained by gradually removing independent variables with the highest *p*-values until only those significant at the 0.05 level remained.

The results from the linear regressions are presented with non-standardized coefficient values (B), 95% confidence intervals (95% CI), and *p*-values. Multiple models were assessed using F statistics, *p*-values, and determination coefficients (R^2^). All the final models met the necessary assumptions: the residuals were normally distributed (as assessed using the Kolmogorov–Smirnov test), no multicollinearity was present (variance inflation factors < 2), and homoscedasticity was observed (indicated by a consistent spread of residuals across the predicted values in the scatter plot).

### 3.4. Ethical Considerations

Ethical approval for this study was obtained from IPPMG (Institute of Pediatrics and Child Health) and CONEP (National Commission in Research Ethics). All pregnant women living with HIV (PWLWHIV) who participated in the study met the inclusion criteria and were thoroughly informed about the study’s objectives. They were made aware that their participation was voluntary, and their responses would remain confidential. Written informed consent was obtained from each participant before data collection began.

Before administering the questionnaires, it was ensured that all the participants understood the information provided and consented to participate, granting permission for the extraction of data for the specified research purposes. The collected data were pseudonymized prior to analysis to protect the participants’ identities. The study adhered to the ethical guidelines outlined in the Declaration of Helsinki for human research.

## 4. Results

This study was performed with 82 PWLWHIV who had vaginal or cesarean deliveries in the public maternity wards of Rio de Janeiro. Table 1 displays the sociodemographic and birth characteristics of the study population.

The participants ranged in age from 19 to 49 years, with a mean age of 28.5 (IQI = [23.3; 28.6]). There were 14 (17.1%) white people, 34 (41.5%) mixed race people, and 34 (41.5%) black people. Almost all of the PWLWHIV had not completed high school education (67–81,8%), and only 2 (2.4%) had completed university education. The majority were married or living unofficially with a partner (58–70.7%), and an additional 24 (29.2%) were single or divorced. Over half (55–67.1%) were employed, and only one was retired (1.2%). Their household incomes ranged from BRL 150 to 900 per month, with a mean of BRL 334.

Their histories ranged from one to six pregnancies, with a mean of one and a half pregnancies. Their parities ranged from zero to five. Forty-two (42–51.2%) were pregnant with their first child. The majority had not received analgesia (57–69.5%). In fact, none of those who had undergone vaginal delivery had received either analgesia or anesthesia during labor and birth. Only those who had undergone cesarean sections had received anesthesia (25–30.5%). Most of the PWLWHIV did not smoke (78–95.1%), drink alcohol (77–93.9%), or use illicit drugs as cocaine (75–92.6%) during pregnancy. Most of the pregnancies were not planned (69–84.1%) or desired (66–80.5%). Almost all the viral loads had a CD4 cell count below 1000 copies/mL at the thirty-fourth week (79–96.3%). Only one was above the detection threshold (1.2%), and two PWLWHIV did not have results. Most of the PWLWHIV had support from companions (78–95.1%).

Regarding demographic characteristics, there were no differences in the responses by race, parity, or socioeconomic status. Unfortunately, it was not possible to analyze in this study the influence of viral load, companion support, planning or desiring the pregnancy, smoking, alcohol or the use of drugs as most of PWLWHIV had the same profile.

Regarding the babies, only six (7.3%) were above gestational age of 41 weeks. The babies were almost evenly divided by gender: 40 female (48.8%) and 42 male (51.2%). The mean weight of babies was 3180 g, ranging from 2580 to 3820 g. The mean length was 50 cm, ranging from 45 to 53 cm. The cephalic perimeter ranged from 31.5 to 39 cm, with mean at 35 cm. There were no differences in demographic characteristics or responses on the study variables by gestational age. Unfortunately, in this study it was not possible to analyze the influence of Apgar score (first and fifth minute) or baby weight because they had the same profile (Table 1). The birth complications in this study were sepsis (4, 4.87%), intrauterine anoxia (2, 2.44%), and respiratory distress syndrome (1, 1.22%).

The PWLWHIV experienced a good total childbirth satisfaction (score133 of 165) and good overall childbirth satisfaction (score 12 out of 15) (Table 2). The PWLWHIV also experienced good self-satisfaction (score 37.3 out of 45), good satisfaction with their partners (score 8.96 out of 10), good satisfaction with midwives (score 35.1 out of 45), and good satisfaction with doctors (score 31.2 out of 45). The PWLWHIV also experienced satisfaction with their babies (score 8.48 out of 10). However, the PWLWHIV could not breastfeed their babies. Therefore, the question about satisfaction with breastfeeding was withdrawn from the questionnaire.

The PWLWHIV’s overall, self, partner, baby, midwives, and doctors satisfaction levels were compared with their feeling of the being birth painful. No statistically significant difference was found, probably due to the very low number of not painful births (only 7) (Table 3).

An initial multiple regression model for each MCSRS subscale was adjusted with all the independent variables that resulted in a *p*-value < 0.02 in the simple models. Then, the non-significant independent variables were removed until only the significant, explicable variables remained in the model. Table 4, Table 5, Table 6, Table 7, Table 8, Table 9 and Table 10 contain the final multiple models and the initial multiple models, which can be found in the Supplementary Materials.

Regarding the final multiple regression model for the total satisfaction with childbirth, vaginal delivery scored 16.43 points higher, on average, than the cesarean section (*p* = 0.008). Additionally, women with birth complications scored 5.86 points lower, on average, than their counterparts (*p* < 0.001). The obstetric history (each delivery) scored 5.19 points higher, on average (*p* = 0.019) (Table 4).

In the results of the multiple regression model for the subscale ‘self’, vaginal delivery scored 4.36 points higher, on average, than the cesarean section (*p* = 0.012). Additionally, women with birth complications scored 1.78 points lower, on average, than their counterparts (*p* < 0.001) (Table 5).

Regarding the multiple regression model for the subscale ‘partner’, occupational status scored 1.08 points lower, on average, than the cesarean section (*p* = 0.017). Additionally, women with birth complications scored 5.86 points lower, on average, than their counterparts (*p* = 0.017). The baby delivered with a male sex scored 0.86 points lower, on average, than female (*p* = 0.039) (Table 6).

In the results of the multiple regression model for the subscale ‘baby’, women with birth complications scored 14.48 points lower, on average, than their counterparts (*p* < 0.001). Vaginal delivery scored 1.67 points higher, on average (*p* < 0.001) (Table 7).

Regarding the multiple regression model for the subscale midwives, women with birth complications scored 5.18 points lower, on average, than their counterparts (*p* < 0.001). The obstetric history (each delivery) scored 1.68 points higher, on average (*p* = 0.020) (Table 8).

Regarding the multiple regression model for the subscale ‘doctors’, women with birth complications scored 13.37 points lower, on average, than their counterparts (*p* < 0.001). Additionally, the male babies scored 3.31 points lower, on average, than the female babies (*p* = 0.034). The obstetric history (each delivery) scored 1.22 points higher, on average (*p* = 0.048) (Table 9).

Regarding the multiple regression model for the overall global satisfaction with childbirth, vaginal delivery scored 1.69 points higher, on average, than the cesarean section (*p* = 0.007). Additionally, women with birth complications scored 5.86 points lower, on average, than their counterparts (*p* < 0.001) (Table 10).

## 5. Discussion

Patient satisfaction is a crucial indicator of the quality of care provided in healthcare settings. In the context of childbirth, evaluating women’s satisfaction is essential for improving obstetric care and ensuring positive outcomes for both mothers and newborns [38].

Childbirth satisfaction is a multidimensional concept, incorporating various aspects of the birth experience. It can reflect satisfaction not only with the birth process itself but also with the support from healthcare providers, the birth environment, and personal well-being.

Research has shown that the most significant predictors of overall satisfaction with childbirth include the birth of a healthy baby, and whether a woman’s expectations for labor and delivery are met. To enhance satisfaction, healthcare providers, such as nurses and doctors, should focus their supportive interventions on aligning with the woman’s expectations, both during prenatal care and throughout labor and delivery [26]. For PWLWHIV, it is especially important to assess and discuss their wishes and expectations throughout pregnancy. This ongoing dialog can help to ensure that a personalized birth plan is developed, addressing PWLWHIV’s unique needs and concerns. Creating a birth plan that aligns with their expectations may help to ensure that PWLWHIV experience greater satisfaction and more positive childbirth experiences.

The PWLWHIV in this study had their labor expectations met and were satisfied. The PWLWHIV experienced good total childbirth satisfaction (scoring 133 out of 165) and good overall childbirth satisfaction (scoring 12 out of 15). Other studies carried out in Brazil using the MCSRS found the same. Ramos et al. found that the average score was 141 points in a sample of 243 postpartum women without HIV, with a standard deviation of 16.8. They also found a high satisfaction among women without HIV virus in all domains of the scale, and a low adherence to currently recommended good labor and birth care practices [39]. The PWLWHIV also experienced a good self-satisfaction (score 37.3 out of 45), good satisfaction with partner (score 8.96 out of 10), good satisfaction with midwives (score 35.1 out of 45), and good satisfaction with doctors (score 31.2 out of 45). The PWLWHIV also experienced satisfaction with their babies (score 8.48 out of 10). These findings align with other studies that have shown a positive relationship between expectations and childbirth satisfaction [40,41]. When a woman’s expectations for childbirth are met, it tends to lead to greater satisfaction with the overall experience. The congruence between what women expect and what they actually experience during childbirth plays a significant role in determining their satisfaction. In many cases, women evaluate their satisfaction based on whether they achieved their anticipated outcomes, feeling a sense of accomplishment when their expectations are fulfilled. This underscores the importance of addressing and aligning expectations throughout pregnancy to enhance the childbirth experience. Therefore, to fully understand women’s satisfaction with childbirth, it is essential to evaluate components of the childbirth experience of PWLWHIV.

PWLWHIV generally expect a range of considerations and interventions aimed at optimizing maternal and fetal outcomes, while minimizing the risk of the mother-to-child transmission (MTCT) of HIV. However, PWLWHIV also expect to have a positive experience at childbirth [42]. Personal control during childbirth, the fulfillment of expectations, and access to preferred pain relief methods were significant predictors of higher satisfaction [8,26,33]. Additionally, living with a partner was associated with higher satisfaction score [8].

Nurses in labor and delivery play a critical role in supporting PWLWHIV by helping them organize and manage both themselves and their environment to meet their expectations and maintain a sense of personal control. This support can be particularly important as it empowers women to navigate the challenges of labor and delivery with greater confidence. If some of the woman’s expectations are not met during the process, it is crucial for the nurse to provide counseling and assist the woman in understanding the circumstances surrounding those unmet expectations. Rather than allowing the woman to blame herself, the nurse should help her accept the aspects of the situation that were beyond her control, promoting emotional resilience and reducing feelings of guilt or frustration. By facilitating the woman’s expectations and encouraging personal control, nurses can enhance the overall birth experience, leading to greater satisfaction. This approach supports not just the physical but also the emotional and psychological well-being of PWLWHIV during labor and delivery, contributing to a more positive and empowering childbirth experience [26,43,44].

The absence of complications with the baby was a good predictor of the satisfaction of PWLWHIV at childbirth in our study, which was in accordance with the literature on HIV-negative pregnant women. Complications such as low Apgar scores and the need for neonatal intensive care unit (NICU) admission are associated with decreased maternal satisfaction with childbirth [45,46,47]. Additionally, obstetric interventions, such as emergency cesarean sections and postpartum hemorrhage, have been linked to lower satisfaction scores. These interventions often correlate with adverse neonatal outcomes, which can further impact maternal perceptions of the childbirth experience [46,48]. Overall, the literature suggests that both the occurrence of neonatal complications and the quality of care during childbirth significantly affect maternal satisfaction. Improving communication, providing adequate support, and minimizing unnecessary interventions may enhance satisfaction and potentially improve neonatal outcomes [46]. On the other hand, unfortunately, PWLWHIV are at increased risk for adverse pregnancy outcomes. These include higher rates of preterm birth, low birth weight, and intrauterine growth restriction. [46] They also experience higher incidences of obstetric complications such as preterm premature rupture of membranes, postpartum sepsis, and venous thromboembolism. Despite the use of antiretroviral therapy (ART), these risks remain elevated compared to those in HIV-negative women [46].

The type of delivery and the obstetric history were also important for PWLWHIV satisfaction. The previous experiences of PWLWHIV could influence their expectations and be reflected in their satisfaction at childbirth. Previous birth experiences, including the mode of delivery and the number of prior births, significantly influence women’s satisfaction with childbirth [49,50,51,52]. These factors can have lasting effects on psychological well-being and relationship dynamics. The mode of delivery plays a significant role in shaping maternal satisfaction. For instance, women who have vaginal deliveries tend to report higher fulfillment and lower distress compared to those who undergo cesarean sections [52]. Additionally, prior deliveries are associated with a lower risk of a negative birth experience, suggesting that experience may contribute to increased satisfaction [51].

The long-term psychological outcomes of childbirth experiences also impact satisfaction. Women maintain clear memories of their childbirth experiences even years later, and these experiences are linked to long-term outcomes such as mental health and sexual satisfaction [50]. Furthermore, the transition to motherhood can affect relationship satisfaction, with first-time mothers experiencing a more pronounced decrease in satisfaction compared to those with subsequent births [49].

In Brazil, significant changes are currently underway in childbirth care, with a focus on reducing the rate of cesarean births and promoting more mother-centered care. The shift emphasizes the active participation of women in the childbirth process, encouraging them to take a more protagonistic role in their own experience. This change aligns with a broader movement to improve the quality of maternal healthcare by prioritizing the well-being, autonomy, and preferences of women. To effectively support and guide these changes, it is essential to understand the perspectives of women who have experienced childbirth. By gathering and analyzing their experiences, healthcare providers can identify areas for improvement and design interventions that better meet women’s needs and expectations. This feedback is crucial for the continuous enhancement of childbirth care, ensuring that it is not only clinically effective but also emotionally and psychologically supportive, contributing to better overall maternal and neonatal outcomes [8].

Many emotional factors influence the childbirth satisfaction of PWLWHIV such as HIV-related stigma, discrimination, the absence of social or family support, violence from intimate partners, and patients’ cultural and social expectations. Stigma and shame associated with HIV can negatively impact the childbirth experience. Women who face stigma within the healthcare system or in their communities may feel reluctant to disclose their HIV status, which can lead to suboptimal adherence to care and reduced social support [53]. The level of support received from partners, family, and friends is crucial. Women with greater social support tend to have more positive attitudes toward pregnancy and childbirth, which improves satisfaction with the experience [54,55]. In our study, almost all the PWLWHIV had partners helping and participating during labor and childbirth. Unfortunately, we could not evaluate the partner’s absence. Symptoms of depression and anxiety are common among pregnant women living with HIV and are associated with worse emotional and health outcomes. Antenatal depression, in particular, can increase the risk of adverse birth outcomes and decrease satisfaction with the experience [56,57]. Intimate partner violence is a significant contributor to emotional distress and can negatively impact satisfaction with childbirth. Women who experience violence are more likely to report depressive symptoms and lower satisfaction with the childbirth experience [56]. The decision whether to disclose one´s HIV status can be a significant source of stress. Concerns about discrimination and judgment can lead to feelings of isolation and anxiety, impacting satisfaction with childbirth [21]. These emotional factors are interrelated and can significantly influence satisfaction with childbirth in pregnant women living with HIV. Interventions that address these emotional aspects, such as psychosocial support and stigma reduction, are essential for improving the birth experiences of these women [21].

According to the study by DLarrabee et al. [19], women with HIV reported increased health distress and worse health transition during antenatal visits compared to HIV-negative controls. During the perinatal period, HIV-negative patients experienced a decreased sense of overall health and worse health transition, suggesting that labor and delivery may be particularly challenging for all women, regardless of HIV status. However, the study does indicate that PWLWHIV generally have a different quality of life trajectory compared to HIV-negative women, with increased health distress and worse health transition during pregnancy and postpartum periods [19].

The present study has limitations. Our sample was small, and some analysis were not statistically possible because the majority of events were equal or similar. The Brazilian version of the MCSRS tool was applied in public hospital at Rio de Janeiro, and the interviewed PWLWHIV were from low-income families. Low socioeconomic status (SES) is often associated with reduced access to high-quality maternity care, which can negatively impact maternal satisfaction. In Malaysia and Kenya, studies found that household income was significantly associated with maternal satisfaction during labor, indicating that lower income may correlate with lower satisfaction levels [58,59]. Similarly, in England, women from lower SES groups reported less respectful treatment and poorer communication from healthcare providers, which could contribute to dissatisfaction [60]. In Brazil, satisfaction with childbirth care was linked to sociodemographic factors, with respect and good practices during childbirth being crucial for satisfaction. This implies that SES-related differences in care quality and respect can influence satisfaction [61]. Socioeconomic disparities affect access to care, quality of interactions with healthcare providers, and the overall childbirth experience, all of which are critical determinants of maternal satisfaction during childbirth. Addressing these disparities through targeted interventions could improve satisfaction across different SES groups.

Another limitation of this study was the number of PWLWHIV who had a low viral load (under 1000 copies per mL) at 34 weeks of gestation. This could have influenced the satisfaction of PWLWHIV childbirth, as low viral load could have lessened birth complications and lessened vertical transmission. From our results, anything that could raise birth complications could interfere then with the outcomes. It would be unethical to conduct a study aiming for high viral loads through not providing antiretroviral medications, as antiretroviral medications must be performed for all PWLWHIV. Another consideration is that a low viral load enables PWLWHIV to choose more details about their childbirth, given that when there is a high viral load, a cesarean section was preferred. As presented in our results, almost all deliveries were vaginal.

## 6. Conclusions

PWLWHIV had a positive experience at childbirth in the public maternity wards of Rio de Janeiro. Our findings indicate that complications with baby health at birth were the main factor associated with a negative experience. As found by other studies the perinatal period is a time of stress for all pregnant women, specially PWLWHIV [45]. 

Challenges faced by pregnant women living with HIV (PWLWHIV) can hinder their successful engagement in HIV care and negatively impact their quality of life, as well as that of their children. These challenges often include stigma, discrimination, mental health issues, and barriers to accessing care and support [21].

To address these challenges effectively, strategies should adopt a broader, structural approach that includes not just the women, but also their partners, family members, and community. It is essential to involve policymakers, funders, and program implementers in these efforts to create a coordinated response. By working together, these stakeholders can help reduce the psychological distress experienced by women living with HIV and create a supportive environment that enables better access to care.

Such consolidated efforts have the potential to create long-lasting, sustainable solutions that benefit not only the women and their families but also the wider community. By addressing the root causes of these challenges and fostering a more supportive and inclusive environment, we can improve health outcomes, reduce stigma, and enhance the overall quality of life for PWLWHIV and their children.

### Recommendations for Future Research

Future research for improving the childbirth satisfaction of PWLWHIV should include approaches involving emotional, social, and health factors. Respectful maternal care should be encouraged and disseminated in order to reduce HIV-related stigma and increase providers’ self-efficacy in caring for PWLWHIV [53,54]. Structural barriers, such as limited access to healthcare and transportation should also be addressed in order to improve a positive experience at childbirth.

Comprehensive social and family support should improve quality of life, reduce anxiety and depression, and increase childbirth satisfaction. One study showed that women who received social and family support had better emotional and health outcomes [21,62].

Peer support through weekly meetings during prenatal and postnatal care could improve the PWLWHIV’s childbirth by providing meaningful emotional support and serving as a complement to professional and family support. Peer support care could offer emotional support and help to reduce feelings of stigma and isolation. It fosters a sense of community and understanding among women who share similar experiences, which can enhance self-confidence and emotional well-being. Peer mentors can reinforce medical advice, particularly regarding the prevention of the mother-to-child transmission (PMTCT) of HIV. This includes promoting adherence to antiretroviral therapy (ART) and exclusive breastfeeding practices, which are crucial for reducing transmission risks [63,64].

Programs involving peer support should be increased as it has been shown that peer support improves retention in HIV care and ART adherence. This is particularly important during the postpartum period, where there is often a decline in care engagement [65,66]. Overall, peer support is a valuable adjunct to professional healthcare services, offering a multidimensional approach to improving outcomes for PWLWHIV and their infants.

Programs should emphasize psychosocial care and incorporate strategies to optimize well-being and satisfaction with childbirth, with a multidisciplinary approach helping PWLWHIV filling up their birth plan as a directive healthcare to avoid stigma and promote decision-making [67].

## Figures and Tables

**Table 1:** Socio-demographic characteristics of the sample (N = 82).

	Descriptive Statistics
Mother	
Maternal age at birth	28.5 [23.3; 28.6], 19–49
Ethnicity	
White	14 (17.1%)
Mixed race	34 (41.5%)
Black	34 (41.5%)
Marital status	
Married	22 (26.8%)
Single or divorced	24 (29.3%)
Consensual union	36 (43.9%)
Education	
Primary + incomplete high school	67 (81.8%)
Complete high school + university	15 (18.2%)
Number of pregnancies	1.5 [1; 3], 1–6
Number of previous deliveries	0 [0; 2], 0–7
Planned pregnancy	
No	69 (84.1%)
Yes	13 (15.9%)
Viral load at 34 weeks	
CD4 cell count higher than 1000 copies/mL	1 (1.2%)
CD4 cell count below 1000 copies/mL	79 (96.3%)
PWLWHIV without viral load at 34 weeks	2 (2.4%)
Smoking	
No	78 (95.1%)
Yes	4 (4.9%)
Alcohol	
No	77 (93.9%)
Yes	5 (6.1%)
Illicit Drugs as cocaine	
No	75 (92.6%)
Yes	6 (7.4%)
Companion at labor/birth	
No	4 (4.9%)
Yes	78 (95.1%)
Monthly income	1890 (549), 800–3500
First baby	
No	40 (48.8%)
Yes	42 (51.2%)
Occupational status	
Retired due to HIV	1 (1.2%)
Unemployed	18 (22.0%)
Housekeeper	5 (6.1%)
Employed	55 (67.1%)
Student	3 (3.7%)
Desired pregnancy	
No	66 (80.5%)
Yes	(19.5%)
Painful birth	
No	7 (8.5%)
Yes	75 (91.5%)
Birth type	
Cesarean	25 (30.5%)
Vaginal	57 (69.5%)
Baby	
Age of baby at delivery	
38 weeks	20 (24.4%)
39 weeks	32 (39.0%)
40 weeks	24 (29.3%)
41 weeks	6 (7.3%)
Sex of baby	
Female	40 (48.8%)
Male	42 (51.2%)
Weight of Baby	3180 (247), 2580–3820
Apgar 1st min	9 [8; 9], 1–9
Apgar 5th min	9 [9; 9], 2–10
Length (cm)	50 [49; 50.8], 45–53
Cephalic perimeter	35 [34; 36], 31.5–39
Birth complications	
No	75 (91.5%)
Yes	7 (8.5%)

Results are described using absolute and relative frequencies (*n* (%)); median [1st quartile; 3rd quartile]; and mean (SD), min–max.

**Table 2:** Descriptives of MCSRS subscales (N = 82).

Subscales	Mean	SD	Median	Min–Max
MCSRS Overall global satisfaction (3 items: 1, 2 and 34)	12.1	3.16	13	3–15
MCSRS self (9 items: 3 to 11)	37.3	9.49	36	9–45
MCSRS partner (2 items: 12 and 13)	8.96	2.01	10	2–10
MCSRS baby * (2 items: 14 and 15)	8.48	2.54	10	2–10
MCSRS midwives (9 items: 17, 19, 21, 23, 25, 27, 29, 31, 33)	35.1	9.23	36	9–45
MCSRS doctors (8 items: 18, 20, 22, 24, 26, 28, 30, 32)	31.2	8.04	32	8–40
MCSRS Total Childbirth Satisfaction	133	31.6	136	33–165

* MCSRS baby do not have item 16 of the original questionnaire because PWLWHIV could not breastfeed.

**Table 3:** Comparison of subscales between painful or not painful births (N = 82).

	Painful Birth (N = 75)	Non-Painful Birth (N = 7)	Mann-Whitney’s *p*-Value
MCSRS Overall global satisfaction	13 [12; 14]	12 [8.5; 14]	0.486
MCSRS self	36 [36; 45]	36 [32.5; 40.5]	0.399
MCSRS partner	10 [8; 10]	10 [8; 10]	0.844
MCSRS baby *	10 [8; 10]	10 [6.5; 10]	0.640
MCSRS midwives	36 [36; 38]	43 [26.5; 45]	0.756
MCSRS doctors	32 [32; 34.5]	26 [23; 40]	0.607
MCSRS Childbirth Satisfaction	136 [132; 147]	122 [106.5; 159.5]	0.681

* MCSRS baby do not have itemn 16 of the original questionnaire as PWLWHIV could not breastfeed.

**Table 4:** Multiple regression analysis for MCSRS of ‘childbirth satisfaction’ (N = 82).

	Beta [95% CI]	*p*-Value
MCSRS Childbirth Satisfaction		
Number of previous deliveries	5.19 [0.88; 9.50]	0.019
Type of birth		
Cesarean	*Reference*	
Vaginal	16.43 [4.42; 28.43]	0.008
Birth complications		
No	*Reference*	
Yes	−58.79 [−78.48; −39.10]	<0.001
	F (3, 78) = 18.4 R^2^ = 0.415	<0.001

**Table 5:** Multiple regression analysis for MCSRS of ‘self’ (N = 82).

	Beta [95% CI]	*p*-Value
MCSRS Self Satisfaction		
Type of birth		
Cesarean	*Reference*	
Vaginal	4.36 [1.00; 7.71]	0.012
Birth complications		
No	*Reference*	
Yes	−21.06 [−26.59; −15.53]	<0.001
	F (2, 79) = 36.7 R^2^ = 0.482	<0.001

**Table 6:** Multiple regression analysis for MCSRS of ‘partner’ (N = 82).

	Beta [95% CI]	*p*-Value
MCSRS Partner Satisfaction		
Occupational status		
Retired + employed	*Reference*	
Others	−1.08 [−1.96; −0.20]	0.017
Baby sex		
Female	*Reference*	
Male	−0.86 [−1.68; −0.05]	0.039
Birth complications		
No	*Reference*	
Yes	−1.78 [−3.24; −0.32]	0.017
	F (3, 78) = 5.93 R^2^ = 0.186	<0.001

**Table 7:** Multiple regression analysis for MCSRS of ‘baby’ (N = 82).

	Beta [95% CI]	*p*-Value
MCSRS Baby Satisfaction		
Type of birth		
Cesarean	*Reference*	
Vaginal	1.67 [0.77; 2.57]	<0.001
Birth complications		
No	*Reference*	
Yes	−5.18 [−6.66; −3.70]	<0.001
	F (2, 78) = 36.6 R^2^ = 0.484	<0.001

**Table 8:** Multiple regression analysis for MCSRS of ‘midwives’ (N = 82).

	Beta [95% CI]	*p*-Value
MCSRS Midwives Satisfaction		
N^er^ of previous deliveries	1.68 [0.28; 3.09]	0.020
Birth complications?		
No	*Reference*	
Yes	−14.48 [−20.84; −8.12]	<0.001
	F (2, 79) = 13.3 R^2^ = 0.252	<0.001

**Table 9:** Multiple regression analysis for MCSRS of ‘doctors’ (N = 82).

	Beta [95% CI]	*p*-Value
MCSRS Doctors Satisfaction		
N^er^ of previous deliveries	1.22 [0.01; 2.43]	0.048
Baby sex		
Female	*Reference*	
Male	−3.31 [−6.36; −0.25]	0.034
Birth complications		
No	*Reference*	
Yes	−13.37 [−18.76; −7.98]	<0.001
	F (3, 78) = 11.4 R^2^ = 0.305	<0.001

**Table 10:** Multiple regression analysis for MCSRS ‘overall global satisfaction’ (N = 82).

	Beta [95% CI]	*p*-Value
MCSRS Overall Global Satisfaction		
Number of previous deliveries	0.44 [0.09; 0.88]	0.046
Baby sex		
Female	*Reference*	
Male	1.69 [0.48; 2.90]	0.007
Birth complications		
No	*Reference*	
Yes	−5.86 [−7.85; −3.88]	<0.001
	F (3, 78) = 17.8 R^2^ = 0.406	<0.001

## Data Availability

All the data could be asked to the corresponding author. It is not publicaly published due to ethical restrictions.

## Supplementary Materials

The following supporting information (Mackey questionnaire adapted to Brazil and all the tables from the sudy including single and mulitples regression analysis) can be downloaded at: https://www.mdpi.com/article/10.3390/jcm14061975/s1.
