# Supplementary material for: The Childbirth Experience of Pregnant Women Living with HIV Virus
Source: J Clin Med. 2025 Mar 14;14(6):1975. doi: 10.3390/jcm14061975 (PMC11943000; doi:10.3390/jcm14061975)
Supplement: Supplementary file 1 [file jcm-14-01975-s001.zip › jcm-3461305-supplementary.pdf]

Mackey questionnaire Brazilian version

MACKEY CHILDBIRTH SATISFACTION RATING SCALE

Caso #

Data de Aplicação ___/___/___

(Por favor, circule o código de uma resposta correta para cada questão, a menos que seja instruída de outra forma).
Estou interessada na sua avaliação sobre sua experiência no parto

Para cada um dos seguintes itens listados abaixo, **indique quão satisfeita ou insatisfeita você está com** esse aspecto de sua experiência no parto. (Circule uma resposta para cada item).

	**Muito insatisfeita**	**Insatisfeita**	**Nem satisfeita ou insatisfeita**	**Satisfeita**	**Muito Satisfeita**
**1**. A sua experiência geral durante o trabalho de parto...	**1**	**2**	**3**	**4**	**5**
**2**. A sua experiência geral durante o parto (o momento em que ocorreu a expulsão do bebê)	**1**	**2**	**3**	**4**	**5**
**3.** A sua participação nas decisões durante o trabalho de parto..	**1**	**2**	**3**	**4**	**5**
**4.** A sua participação nas decisões durante o parto (o momento em que ocorreu a expulsão do bebê)...	**1**	**2**	**3**	**4**	**5**
**5.** A sua capacidade de lidar com as contrações durante o trabalho de parto............	**1**	**2**	**3**	**4**	**5**
**6.** O seu conforto ou bem-estar geral durante o trabalho de parto..	**1**	**2**	**3**	**4**	**5**
**7.** O seu conforto ou bem-estar geral durante o parto (o momento em que ocorreu a expulsão do bebê)...	**1**	**2**	**3**	**4**	**5**

**8.** O controle que você teve sobre suas emoções durante o trabalho de parto.............	**1**	**2**	**3**	**4**	**5**
**9.** O controle que você teve sobre suas emoções durante o parto (o momento em que ocorreu a expulsão do bebê)...........................	**1**	**2**	**3**	**4**	**5**
**10.** O controle que você teve sobre suas ações durante o trabalho de parto...............................	**1**	**2**	**3**	**4**	**5**
**11. O controle que você teve sobre suas ações** durante o parto (o momento em que ocorreu a expulsão do bebê)...	**1**	**2**	**3**	**4**	**5**
12. A colaboração do seu marido/companheiro durante o trabalho de parto..............................	1	2	3	4	5
13. A colaboração do seu marido/companheiro durante o parto (o momento em que ocorreu a expulsão do bebê)..	1	2	3	4	5
14. As condição físicas e de saúde do seu bebê no nascimento..	1	2	3	4	5
15. O tempo que demorou para você segurar seu bebê pela 1ª vez.......................................	1	2	3	4	5
16. O tempo que demorou para você amamentar seu bebê pela 1ª vez....................	1	2	3	4	5
17. Os cuidados com o seu corpo que recebeu da equipe de enfermagem durante o trabalho de parto e o parto...	1	2	3	4	5
18. Os cuidados com o seu corpo que recebeu da equipe médica durante o trabalho de parto e o parto...	1	2	3	4	5
19. O conhecimento técnico, a habilidade e competência da equipe de enfermagem durante o trabalho de parto e o parto..............	1	2	3	4	5

20. O conhecimento técnico, a habilidade e competência da equipe médica durante o trabalho de parto e o parto...............................	1	2	3	4	5
21. A quantidade de explicações ou informações que você recebeu da equipe de enfermagem durante o trabalho de parto e o parto................	1	2	3	4	5
22. A quantidade de explicações ou informações que você recebeu da equipe médica durante o trabalho de parto e o parto..	1	2	3	4	5
23. O interesse pessoal da equipe de enfermagem e a atenção dada a você durante o trabalho de parto e o parto............................	1	2	3	4	5
24. O interesse pessoal da equipe médica e a atenção dada a você durante o trabalho de parto e o parto...	1	2	3	4	5
25. A ajuda da equipe de enfermagem e apoio com a respiração e o relaxamento que você recebeu durante o trabalho de parto e o parto	1	2	3	4	5
26. A ajuda da equipe médica e apoio com a respiração e o relaxamento que você recebeu durante o trabalho de parto e o parto..............	1	2	3	4	5
27. O tempo que os enfermeiros (as) dedicaram a você durante o trabalho de parto	1	2	3	4	5
28. O tempo que os médicos (as) dedicaram a você durante o trabalho de parto.....................	1	2	3	4	5
29. A atitude dos enfermeiros (as) durante o trabalho de parto e no parto............................	1	2	3	4	5
30. A atitude dos médicos (as) durante o trabalho de parto e no parto............................	1	2	3	4	5
31. A sensibilidade da equipe de enfermagem às suas necessidades, durante o trabalho de parto e no parto..	1	2	3	4	5

32. A sensibilidade da equipe médica às suas necessidades, durante o trabalho de parto e no parto..	1	2	3	4	5
33. Sua satisfação geral sobre os cuidados que você recebeu durante o trabalho de parto e no parto..	1	2	3	4	5
34. No geral, qual o seu grau de satisfação ou insatisfação com a sua experiência no nascimento do seu bebê?...............................	1	2	3	4	5

35. Anote o que contribuiu para a sua satisfação / insatisfação geral com a sua experiência no nascimento do seu bebê.

_ _ _ _ _ _ _ _ _ _ _ _ _ _
_ _ _ _ _ _ _ _ _ _ _ _ _ _ _
_ _ _ _ _ _ _ _ _ _ _ _ _ _ _
_ _ _ _ _ _ _ _ _ _ _ _ _ _ _

36. Usando os itens que você anotou na questão 35 acima, numere-os em ordem de importância: Coloque "1" na frente do item que mais contribuiu para a sua satisfação / insatisfação; coloque "2" a partir do próximo item mais importante e assim por diante até numerar todos os itens.

37. De forma geral, a sua experiência no trabalho de parto foi como você esperava? **(Circule uma resposta).**
Nada a ver com o que eu esperava 1
Muito pouco a ver com o que eu esperava ... 2
Um pouco a ver com o que eu esperava 3
Foi como eu esperava 4

38. De forma geral, a sua experiência no parto (o momento em que ocorreu a expulsão do bebê) foi como você esperava? **(Circule uma resposta).**
Nada a ver com o que eu esperava 1
Muito pouco a ver com o que eu esperava ... 2 Um pouco a ver com o que eu esperava 3
Foi como eu esperava 4

39. No geral, você avaliaria o seu trabalho de parto como sendo essencialmente uma experiência positiva ou negativa? **(Circule uma resposta).**

Foi muito negativa 1
Foi um pouco negativa.................................. 2
Foi um pouco positiva 3
Foi muito positiva 4

40. No geral, você avaliaria o seu parto como sendo essencialmente uma experiência positiva ou negativa? **(Circule uma resposta).**

Foi muito negativa 1

Foi um pouco negativa.................................. 2
Foi um pouco positiva 3
Foi muito positiva 4

Suplementary tables

Table S1 Simple regression for MCSRS Childbirth Satisfaction (N=82)

	Beta [IC 95%]	p-value
Maternal age	0.52 [-0.61; 1.64]	0.365
Race		
White	*Reference*	
Mixed	0.63 [-19.5; 20.8]	0.950
black	5.92 [-14.2; 26.1]	0.560
obst_hist_para (number of births)	4.88 [-0.54; 10.3]	0.077
Civil status		
Married	*Reference*	
Single ou divorcede	-6.20 [-24.9; 12.5]	0.512
Consensua unionl	-4.44 [-21.6; 12.7]	0.608
monthly_income	-0.007 [-0.020; 0.006]	0.291
escolarity		
Elementar + high school incomplete	*Reference*	
High school complete + university	-7.87 [-21.7; 5.99]	0.262
Ocupational status		
Employed + retired	*Reference*	
Others	-12.8 [-27.5; 1.96]	0.088
desired_pregnancy		
No	*Reference*	
Yes	7.23 [-11.8; 26.3]	0.452
Painful birth		

No	*Reference*	
Yes	3.91 [-21.1; 28.9]	0.756
Duration of labor (hours)	0.03 [-0.47; 0.53]	0.906
Type of birth		
Cesarean	*Reference*	
Vaginal	21.4 [6.98; 35.8]	**0.004**
Baby sex		
Female	*Reference*	
Male	-11.7 [-25.4; 2.05]	0.094
apgar 1ºmin	7.99 [4.08; 11.9]	**<0.001**
first baby		
No	*Reference*	
Yes	-8.55 [-22.4; 5.29]	0.223
Birth complications		
No	*Reference*	
Yes	-64.0 [-84.6; -43.5]	**<0.001**
Live with baby´s father		
No	*Reference*	
Yes	0.96 [-20.4; 22.3]	0.929

Table S2 Multiple Regression initial and final for MCSRS Childbirth Satisfaction (N=82)

	**Initial Model** $F(6,75) = 9.76; p<0.001; R^2 = 0.438$		**Final Model** $F(3, 78) = 18.4; p<0.001; R^2 = 0.415$	
	**Beta [IC 95%]**	**p-value**	**Beta [IC 95%]**	**p-value**
obst_history_para (birth number)	4.39 [-0.01; 8.80]	0.050	5.19 [0.88; 9.50]	**0.019**
Ocupational status				
Employed + retired	*Reference*			

Others	4.51 [-18.84; 27.86]	0.702		
Type of birth				
Cesarean	*Reference*		*Reference*	
Vaginal	18.04 [-6.25; 42.34]	0.143	16.43 [4.42; 28.43]	**0.008**
Baby sex				
Feminino	*Reference*			
Masculino	-8.78 [-20.12; 2.56]	0.127		
apgar 1ºmin	0.94 [-3.25; 5.13]	0.657		
Birth complications				
Não	*Reference*		*Reference*	
Sim	-56.58 [-80.68; -32.47]	<0.001	-58.79 [-78.48; -39.10]	**<0.001**

Table S3 Simple Regression for MCSRS Self (N=82)

	Beta [IC 95%]	p-value
Maternal age	0.13 [-0.21; 0.47]	0.446
Race		
White	*Reference*	
Mixed	-1.68 [-7.73; 4.37]	0.583
black	-0.06 [-6.11; 5.99]	0.985
obst_hist_para (number of births)	0.98 [-0.67; 2.62]	0.243
Civil status		
Married	*Reference*	
Single ou divorcede	-1.05 [6.68; 4.59]	0.713
Consensua unionl	-1.05 [-6.21; 4.12]	0.688
monthly_income	-0.002 [-0.006; 0.002]	0.318
escolarity		
Elementar + high school incomplete	*Reference*	
High school complete + university	-2.48 [-6.64; 1.68]	0.239
Ocupational status		

Employed + retired	*Reference*	
Others	-3.90 [-8.33; 0.52]	0.083
desired_pregnancy		
No	*Reference*	
Yes	2.87 [-2.84; 8.57]	0.321
Painful birth		
No	*Reference*	
Yes	1.09 [-6.42; 8.59]	0.774
Duration of labor (hours)	-0.04 [-0.19; 0.11]	0.568
Type of birth		
Cesarean	*Reference*	
Vaginal	6.62 [2.31; 10.9]	**0.003**
Baby sex		
Female	*Reference*	
Male	-2.04 [-6.21; 2.13]	0.334
apgar 1ºmin	2.76 [1.62; 3.90]	**<0.001**
first baby		
No	*Reference*	
Yes	-1.94 [-6.12; 2.23]	0.357
Birth complications		
No	*Reference*	
Yes	-22.3 [-28; -16.7]	**<0.001**
Live with baby´s father		
No	*Reference*	
Yes	0.32 [-6.09; 6.73]	0.921

Table S4 Multiple Regression initial and final for MCSRS Self (N=82)

	**Modelo Initial**	**Modelo Final**
	**F(4, 77) = 18.7; p<0.001;**	**F(2, 79) = 36.7; p<0.001;**
	**R^2 = 0.492**	**R^2 =0.482**

	Beta [IC 95%]	p-value	Beta [IC 95%]	p-value
Occupational status				
Retired + employed	*Reference*			
others	2.40 [-4.13; 8.91]	0.467		
Type of birth				
cesarean	*Reference*		*Reference*	
vaginal	6.25 [-0.49; 12.98]	0.069	4.36 [1.00; 7.71]	**0.012**
apgar 1ºmin	0.55 [-0.63; 1.72]	0.358		
Birth complications				
No	*Reference*		*Reference*	
Yes	-18.96 [-25.69; -12.24]	<0.001	-21.06 [-26.59; -15.53]	**<0.001**

Table S5 Simple Regression for MCSRS Partner (N=82)

	Beta [IC 95%]	p-value
Maternal age	0.03 [-0.04; 0.10]	0.447
Race		
White	*Reference*	
Mixed	0.12 [-1.15; 1.39]	0.849
black	0.65 [-0.62; 1.92]	0.312
obst_hist_para (number of births)	0.16 [-0.19; 0.51]	0.368
Civil status		
Married	*Reference*	
Single ou divorcede	-0.91 [-2.09; 0.27]	0.127
Consensua unionl	-0.51 [-1.59; 0.57]	0.350
monthly_income	-0.0003 [-0.001; 0.0005]	0.445
escolarity		
Elementar + high school incomplete	*Reference*	
High school complete + university	-0.07 [-0.96; 0.82]	0.873
Ocupational status		

Employed + retired	*Reference*	
Others	-1.24 [-2.16; -0.33]	**0.008**
desired_pregnancy		
No	*Reference*	
Yes	-0.23 [-1.45; 0.98]	0.706
Painful birth		
No	*Reference*	
Yes	0.43 [-1.16; 2.02]	0.592
Duration of labor (hours)	0.02 [-0.01; 0.05]	0.236
Type of birth		
Cesarean	*Reference*	
Vaginal	1.79 [0.91; 2.67]	**<0.001**
Baby sex		
Female	*Reference*	
Male	-0.90 [-1.77; -0.04]	**0.042**
apgar 1ºmin	0.27 [0.002; 0.54]	**0.048**
first baby		
No	*Reference*	
Yes	-0.32 [-1.20; 0.57]	0.481
Birth complications		
No	*Reference*	
Yes	-1.83 [-3.37; -0.30]	**0.020**
Live with baby´s father		
No	*Reference*	
Yes	0.64 [-0.71; 1.99]	0.347

Table S6 Multiple Regression initial and final for MCSRS Partner (N=82)

	Modelo Inicial	Modelo Final

	$F(7, 74) = 3.97; p<0.001; R^2 = 0.273$		$F(3, 78) = 5.93; p=0.001; R^2 = 0.186$	
	**Beta [IC 95%]**	**p-value**	**Beta [IC 95%]**	**p-value**
Civil status				
Married	*Reference*			
Single or divorced	-0.97 [-2.06; 0.12]	0.080		
Consensual union	-0.67 [-1.71; 0.37]	0.202		
Occupational status				
Retired + employed	*Reference*		*Reference*	
Others	0.38 [-1.36; 2.12]	0.668	-1.08 [-1.96; -0.20]	**0.017**
Type of birth				
cesarean	*Reference*			
vaginal	1.78 [-0.03; 3.60]	0.054		
Baby sex				
Female	*Reference*		*Reference*	
Male	-0.78 [-1.60; 0.05]	0.066	-0.86 [-1.68; -0.05]	**0.039**
apgar 1ºmin	-0.04 [-0.36; 0.27]	0.777		
Birth complications				
No	*Reference*		*Reference*	
Yes	-1.70 [-3.53; 0.12]	0.067	-1.78 [-3.24; -0.32]	**0.017**

Table S7 Simple Regressions for MCSRS Baby (N=82)

	**Beta [IC 95%]**	**p-value**
Maternal age	0.06 [-0.03; 0.15]	0.185
Race		
White	*Reference*	
Mixed	-0.15 [-1.78; 1.48]	0.858
black	0.05 [-1.59; 1.68]	0.956
obst_hist_para (number of births)	0.09 [-0.35; 0.54]	0.676
Civil status		

Married	*Reference*	
Single ou divorcede	-0.10 [-1.63; 1.43]	0.894
Consensua unionl	-0.28 [-1.11; 1.66]	0.694
monthly_income	-0.0007 [-0.002; 0.0003]	0.163
escolarity		
Elementar + high school incomplete	*Reference*	
High school complete + university	-0.31 [-0.44; 0.83]	0.593
Ocupational status		
Employed + retired	*Reference*	
Others	-1.64 [-2.79; -0.48]	**0.006**
desired_pregnancy		
No	*Reference*	
Yes	0.19 [-1.36; 1.73]	0.808
Painful birth		
No	*Reference*	
Yes	0.19 [-1.83; 2.21]	0.854
Duration of labor (hours)	0.004 [-0.04; 0.04]	0.846
Type of birth		
Cesarean	*Reference*	
Vaginal	2.22 [1.10; 3.35]	**<0.001**
Baby sex		
Female	*Reference*	
Male	-0.85 [-1.97; 0.27]	0.135
apgar 1ºmin	0.64 [0.33; 0.96]	**<0.001**
first baby		
No	*Reference*	
Yes	0.14 [-1.00; 1.27]	0.808
Birth complications		
No	*Reference*	
Yes	-5.66 [-7.23; -4.09]	**<0.001**

Live with baby´s father			
	No	*Reference*	
	Yes	0.41 [-1.32; 2.13]	0.639

Table S8 Multiple Regressions initial and final for MCSRS Baby (N=82)

	Modelo Inicial F(7, 73) = 10.8; p<0.001; R^2 = 0.509		Modelo Final F(2, 78) = 36.6; p=0.001; R^2 =0.484	
	**Beta [IC 95%]**	**p-value**	**Beta [IC 95%]**	**p-value**
Maternal age	0.01 [-0.06; 0.09]	0.687		
Monthly income	-4e-5 [-8.6e-4; 7.8e-4]	0.923		
Occupational status				
Retired + employed	*Reference*			
Others	-0.24 [-2.11; 1.62]	0.795		
Type of birth				
Cesarean	*Reference*		*Reference*	
Vaginal	1.27 [-0.67; 3.22]	0.196	1.67 [0.77; 2.57]	**<0.001**
Baby sex				
Female	*Reference*			
Male	-0.77 [-1.65; 0.11]	0.087		
apgar 1ºmin	0.02 [-0.30; 0.35]	0.891		
Birth complications				
No	*Reference*		*Reference*	
Yes	-5.22 [-7.13; -3.31]	<0.001	-5.18 [-6.66; -3.70]	**<0.001**

Table S9 Simple Regression for MCSRS Midwives (N=82)

	**Beta [IC 95%]**	**p-value**
Maternal age	0.15 [-0.18; 0.48]	0.378

Race		
White	*Reference*	
Mixed	1.93 [-3.94; 7.80]	0.515
black	2.96 [-2.91; 8.83]	0.319
obst_hist_para (number of births)	1.75 [0.18; 3.32]	**0.030**
Civil status		
Married	*Reference*	
Single ou divorcede	-1.64 [-7.12; 3.83]	0.552
Consensua unionl	-1.48 [-6.50; 3.55]	0.560
monthly_income	-0.002 [-0.005; 0.002]	0.395
escolarity		
Elementar + high school incomplete	*Reference*	
High school complete + university	-2.01 [-6.07; 2.05]	0.329
Ocupational status		
Employed + retired	*Reference*	
Others	-2.17 [-6.53; 2.19]	0.325
desired_pregnancy		
No	*Reference*	
Yes	1.07 [-4.51; 6.66]	0.703
Painful birth		
No	*Reference*	
Yes	-0.52 [-7.83; 6.79]	0.888
Duration of labor (hours)	0.004 [-0.14; 0.15]	0.954
Type of birth		
Cesarean	*Reference*	
Vaginal	4.23 [-0.11; 8.56]	0.056
Baby sex		
Female	*Reference*	
Male	-3.32 [-7.34; 0.69]	0.104

apgar 1ºmin		1.73 [0.53; 2.92]	**0.005**
first baby			
	No	*Reference*	
	Yes	-3.03 [-7.06; 1.00]	0.138
Birth complications			
	No	*Reference*	
	Yes	-14.6 [-21.2; -8.09]	**<0.001**
Live with baby´s father			
	No	*Reference*	
	Yes	-1.14 [-7.38; 5.09]	0.717

Table S10 Multiple Regression initial and final for MCSRS Midwives (N=82)

		**Modelo Initial** $F(6, 75) = 5.32; p<0.001;$ $R^2 = 0.299$		**Modelo Final** $F(2, 79) = 13.3; p<0.001;$ $R^2 = 0.252$	
		**Beta [IC 95%]**	**p-value**	**Beta [IC 95%]**	**p-value**
obst_hist_para (nº of births)		1.27 [-0.81; 3.35]	0.227	1.68 [0.28; 3.09]	**0.020**
Type of birth					
	cesarean	*Reference*			
	vaginal	2.64 [-1.39; 6.68]	0.196		
Baby sex					
	Female	*Reference*			
	Male	-2.68 [-6.38; 1.01]	0.152		
apgar 1ºmin		0.07 [-1.29; 1.43]	0.920		
1o baby					
	No	*Reference*			
	Yes	-1.15 [-6.32; 4.02]	0.659		
Birth complications					

No	*Reference*		*Reference*	
Yes	-13.87 [-21.73; -6.00]	<0.001	-14.48 [-20.84; -8.12]	**<0.001**

Table S11 Simple Regression for MCSRS Doctors (N=82)

		Beta [IC 95%]	p-value
Maternal age		0.12 [-0.17; 0.41]	0.398
Race			
	White	*Reference*	
	Mixed	1.05 [-4.07; 6.18]	0.683
	black	2.17 [-2.95; 7.30]	0.401
obst_hist_para (number of births)		1.49 [0.12; 2.86]	0.033
Civil status			
	Married	*Reference*	
	Single ou divorcede	-1.81 [-6.57; 2.96]	0.453
	Consensua unionl	-1.04 [-5.41; 3.33]	0.636
monthly_income		-0.001 [-0.005; 0.002]	0.443
escolarity			
	Elementar + high school incomplete	*Reference*	
	High school complete + university	-1.91 [-5.45; 1.62]	0.284
Ocupational status			
	Employed + retired	*Reference*	
	Others	-2.37 [-6.16; 1.42]	0.217
desired_pregnancy			
	No	*Reference*	
	Yes	2.24 [-2.61; 7.08]	0.361
Painful birth			
	No	*Reference*	

Yes	1.62 [-4.74; 7.97]	0.614
Duration of labor (hours)		
Type of birth		
Cesarean	*Reference*	
Vaginal	4.31 [0.57; 8.05]	**0.025**
Baby sex		
Female	*Reference*	
Male	-3.43 [-6.9; 0.05]	0.053
apgar 1ºmin	1.71 [0.69; 2.74]	**0.001**
first baby		
No	*Reference*	
Yes	-2.60 [-6.11; 0.92]	0.145
Birth complications		
No	*Reference*	
Yes	-13.2 [-18.8; -7.53]	**<0.001**
Live with baby´s father		
No	*Reference*	
Yes	0.45 [-4.98; 5.88]	0.870

Table S12 Multiple Regression initial and final for MCSRS Doctors (N=82)

	**Modelo Initial** $F(6, 75) = 6.25; p<0.001; R^2 = 0.333$		**Modelo Final** $F(3, 78) = 11.4; p<0.001; R^2 = 0.305$	
	**Beta [IC 95%]**	**p-value**	**Beta [IC 95%]**	**p-value**
Obst_hist_para (nº of birth)	1.00 [-0.77; 2.76]	0.263	1.22 [0.01; 2.43]	0.048
Type of birth				
Cesarean	*Reference*			
Vaginal	2.72 [-0.71; 6.14]	0.119		
Baby sex				

Female	*Reference*		*Reference*	
Male	-2.77 [-5.90; 0.37]	0.083	-3.31 [-6.36; -0.25]	0.034
apgar 1ºmin	0.28 [-0.88; 1.43]	0.636		
1o baby				
No	*Reference*			
Yes	-1.18 [-5.57; 3.21]	0.595		
Birth complications				
No	*Reference*		*Reference*	
Yes	-11.73 [-18.41; -5.05]	<0.001	-13.37 [-18.76; -7.98]	<0.001

Table S13 Simple Regression for MCSRS Overall Global Satisfaction (N=82)

	**Beta [IC 95%]**	**p-value**
Maternal age	0.02 [-0.09; 0.14]	0.664
Race		
White	*Reference*	
Mixed	-0.65 [-2.66; 1.36]	0.521
black	0.11 [-1.90; 2.12]	0.911
obst_hist_para (number of births)	0.41 [-0.14; 0.96]	0.138
Civil status		
Married	*Reference*	
Single ou divorcede	-0.76 [-2.63; 1.11]	0.421
Consensua unionl	-0.64 [-2.35; 1.08]	0.463
monthly_income	-0.0009 [-0.002; 0.0003]	0.143
escolarity		
Elementar + high school incomplete	*Reference*	
High school complete + university	-1.06 [-2.43; 0.32]	0.131
Ocupational status		
Employed + retired	*Reference*	

Others	-1.44 [-2.90; 0.03]	0.055
desired_pregnancy		
No	*Reference*	
Yes	1.12 [-0.78; 3.02]	0.244
Painful birth		
No	*Reference*	
Yes	1.08 [-1.41; 3.57]	0.389
Duration of labor (hours)	-0.005 [-0.06; 0.04]	0.826
Type of birth		
Cesarean	*Reference*	
Vaginal	2.21 [0.77; 3.64]	**0.003**
Baby sex		
Female	*Reference*	
Male	-1.10 [-2.48; 0.27]	0.114
apgar 1ºmin	0.88 [0.50; 1.26]	**<0.001**
first baby		
No	*Reference*	
Yes	-0.76 [-2.15; 0.63]	0.277
Birth complications		
No	*Reference*	
Yes	-6.39 [-8.45; -4.34]	**<0.001**
Live with baby´s father		
No	*Reference*	
Yes	0.27 [-1.87; 2.40]	0.804

Table S14 Multiple Regression initial and final for MCSRS Overall Global Satisfaction (N=82)

	Modelo Initial	Modelo Final
	**F(8, 73) = 7.09; p<0.001;**	**F(3, 78) = 17.8; p<0.001;**

	R^2 = 0.437		R^2 =0.406	
	**Beta [IC 95%]**	**p-value**	**Beta [IC 95%]**	**p-value**
obst_hist_para (nº of births)	0.35 [-0.12; 0.82]	0.145	0.44 [0.09; 0.88]	0.046
monthly_incomel	-0.0002 [-0.0001; 0.001]	0.807		
escolarity				
Elementar + high school incomplete	*Reference*			
High school complete + university	-0.27 [-1.66; 1.11]	0.695		
Occupational status				
Retired + employed	*Reference*			
Others	0.18 [-2.30; 2.66]	0.883		
Type of birth				
cesarean	*Reference*		*Reference*	
vaginal	1.60 [-0.95; 4.15]	0.215	1.69 [0.48; 2.90]	0.007
Baby sex				
Female	*Reference*			
Male	-0.75 [-1.93; 0.43]	0.209		
apgar 1ºmin	0.23 [-0.20; 0.66]	0.295		
Birth complications				
No	*Reference*		*Reference*	
Yes	-5.10 [-7.62; -2.58]	<0.001	-5.86 [-7.85; -3.88]	<0.001

Table S15 Correlations (Spearman) between MCRS_37, 38, 39 e 40, Total Childbirth Satisfaction e Overall Global Satisfaction (N=82)

**Items**	**MCRS_38**	**MCRS_39**	**MCRS_40**	**MCSRS Childbirth Satisfaction**	**MCSRS Overall global satisfaction**
**MCRS_37**[a]	0.999	0.997	0.997	0.686	0.685
**MCRS_38**	-	0.999	0.999	0.682	0.680
**MCRS_39**	-	-	1	0.685	0.681
**MCRS_40**	-	-	-	0.685	0.681

**MCSRS Childbirth Satisfaction**	-	-	-	-	0.935

p<0.001

Nota: as respostas a MCRS_37, 38, 39 e 40 foram quase sempre as mesmas, daí que a correlação seja muito próxima de 1.

Table S16 Correlations (Spearman) between MCRS_37, 38, 39, 40 and MCRS subscales (N=82)

Items	MCRS_38	MCRS_39	MCRS_40	MCSRS Overall global satisfaction	MCSRS Self	MCSRS Partner	MCSRS Baby	MCSRS Midwives	MCSRS Doctors	MCSRS Total Childbirth Satisfaction
**MCRS_37**[a]	0.999	0.997	0.997	0.685	0.695	0.521	0.734	0.593	0.638	0.686
**MCRS_38**	-	0.999	0.999	0.680	0.687	0.526	0.734	0.592	0.637	0.682
**MCRS_39**	-	-	-	0.681	0.686	0.539	0.736	0.605	0.646	0.685
**MCRS_40**	-	-	-	0.681	0.686	0.539	0.736	0.605	0.646	0.685
**MCSRS Overall global satisfaction**	-	-	-	-	0.934	0.498	0.672	0.757	0.825	0.936
**MCSRS Self**	-	-	-	-	-	0.494	0.681	0.607	0.665	0.871
**MCSRS Partner**	-	-	-	-	-	-	0.815	0.590	0.592	0.662
**MCSRS Baby**	-	-	-	-	-	-	-	0.566	0.662	0.778
**MCSRS Midwives**	-	-	-	-	-	-	-	-	0.921	0.856
**MCSRS Doctors**	-	-	-	-	-	-	-	-	-	0.910

[a] – 1 missing; p<0.001 in all the correlations
